# Subclinical Signs of Retinal Involvement in Hereditary Angioedema

**DOI:** 10.3390/jcm10225415

**Published:** 2021-11-19

**Authors:** Paola Triggianese, Matteo Di Marino, Carolina Nesi, Elisabetta Greco, Stella Modica, Maria Sole Chimenti, Paola Conigliaro, Raffaele Mancino, Carlo Nucci, Massimo Cesareo

**Affiliations:** 1 Rheumatology, Allergology and Clinical Immunology, Department of “Medicina dei Sistemi”, University of Rome Tor Vergata, 00173 Rome, Italy; paola.triggianese@gmail.com (P.T.); elisabetta_greco@yahoo.it (E.G.); stella.modica@ptvonline.it (S.M.); maria.sole.chimenti@uniroma2.it (M.S.C.); paola.conigliaro@uniroma2.it (P.C.); 2 Ophthalmology Unit, Department of Experimental Medicine, University of Rome Tor Vergata, 00173 Rome, Italy; nesi.carolina@gmail.com (C.N.); mancino@med.uniroma2.it (R.M.); nucci@med.uniroma2.it (C.N.); massimo.cesareo@uniroma2.it (M.C.)

**Keywords:** complement system, hereditary angioedema, retina, OCT, OCT-A

## Abstract

To explore retinal abnormalities using spectral domain optical coherence tomography (SD-OCT) and OCT-angiography (OCT-A) in a highly selective cohort of patients with type I hereditary angioedema (HAE). This prospective case-control study included 40 type I HAE patients and 40 age-/sex-matched healthy subjects (HC). All participants underwent SD-OCT-scanning of retinal posterior pole (PP), peripapillary retinal nerve fiber layer (pRNFL), and optic nerve head (ONH). Superficial/deep capillary density was analyzed by OCT-A. A total of 80 eyes from 40 HAE and 40 eyes from HC were evaluated. The pRNFL was thicker in HAE than in HC in nasal superior (*p* < 0.0001) and temporal quadrants (*p* = 0.0005 left, *p* = 0.003 right). The ONH thickness in HAE patients was greater than in HC in the nasal (*p* = 0.008 left, *p* = 0.01 right), temporal (*p* = 0.0005 left, *p* = 0.003 right), temporal inferior (*p* = 0.007 left, *p* = 0.0008 right), and global (*p* = 0.005 left, *p* = 0.007 right) scans. Compared to HC, HAE showed a lower capillary density in both superficial (*p* = 0.001 left, *p* = 0.006 right) and deep (*p* = 0.008 left, *p* = 0.004 right) whole images, and superficial (*p* = 0.03 left) and deep parafoveal (*p* = 0.007 left, *p* = 0.005 right) areas. Our findings documented subclinical retinal abnormalities in type I HAE, supporting a potential role of the retinal assessment by SD-OCT/OCT-A as a useful tool in the comprehensive care of HAE patients.

## 1. Introduction

Hereditary angioedema (HAE; Online Mendelian Inheritance in Man (OMIM) #106100) due to deficiency (type 1) or dysfunction (type II) of C1 esterase inhibitor protein (C1INH) is a rare disorder resulting from the congenital deficiency inherited in an autosomal dominant form [1,2]. The majority of patients show type I HAE resulting from different mutations in the C1INH gene [3,4]. HAE due to C1-INH deficiency is characterized by potentially fatal attacks of subcutaneous and submucosal edema of the upper airways, face, abdomen, and extremities [5]. Frequency and sites of attacks vary widely among HAE patients, often occurring without known triggers [6]. Overall data support the idea that clinical symptoms of C1INH deficiency are attributable to contact system-dependent bradykinin formation rather than complement-related disease mediators [7,8,9,10,11]. Studies concerning molecular mechanisms of vascular permeability in the context of HAE highlighted the key role of kallikrein and bradykinin in vascular endothelial cells activation and stability and edema formation [12,13,14,15]. Vascular endothelial growth factors (VEGFs) and angiopoietins are part of a long list of mediators that have been implicated in endothelial barrier failure in HAE [16,17,18,19]. The increase in bradykinin levels is a key factor in the development of retinopathy (such as diabetic retinopathy) through enhanced vascular permeability, as well as inflammatory mechanisms [20,21]. These deleterious effects are mediated by kinin B1 and B2 receptors, which are expressed in human retina and have a cross-talk with cytokines in promoting inflammation [22,23]. How complement activation at the immune-privileged site of subretinal space is modulated and why the activation becomes detrimental in macular diseases is not known [24,25,26,27].

To date, the swelling of the eyelids represents the only ocular manifestation of HAE [28], while the prevalence of retinal changes has not been thoroughly investigated in HAE. Routine screening of retinal abnormalities is not recommended for individuals with HAE; only patients undergoing prophylactic antifibrinolytic treatment should receive annual ophthalmologic examinations in order to reveal any possible toxic damages caused by the therapy [29].

We recently documented, for the first time, subclinical abnormalities in the retinal microvascular network in type I HAE that could be related to early subtle functional changes [30].

Starting from that intriguing evidence, we aimed at exploring morphologic retinal status from a highly selective cohort of type I HAE patients by using new spectral-domain optical coherence tomography (SD-OCT) [31,32,33]. Furthermore, retinal capillary plexi have been analyzed using OCT-angiography (OCT-A) from all the subjects in the study [30].

## 2. Materials and Methods

In this prospective, case-control study, 40 patients with an established diagnosis of type I HAE who were referred to the tertiary center for HAE (“Policlinico Tor Vergata”, Rome, Italy), were compared with 40 HCs [6].

Inclusion criteria were: (1) diagnosis of type I HAE with subcutaneous non-inflammatory self-limiting angioedema, and with both low serum levels of C1INH antigen and C1-INH function below 50% of normal [25,26,30]; (2) age ≥ 18 years old and ≤75 years old; (3) intraocular pressure (IOP) < 21 mmHg on diurnal testing with measurements using Goldmann applanation tonometry; (4) best-corrected visual acuity (BCVA) ≥ 0.5 logMAR; (5) spherical equivalent refractive error between −6.0 and +4.0 diopters [30,34]. Exclusion criteria were: (1) established primary ocular diseases including ocular trauma or surgery; (2) systemic disorders with ocular involvement such as diabetes and hypertension (current and past medical history); (3) renal dysfunctions; (4) other autoimmune systemic diseases; (5) pregnancy or lactation; (6) neoplasia; and (7) systemic treatments that may affect retinal function. Subjects were also excluded if they presented with a glaucomatous optic nerve configuration [30,32,34].

The control group consisted of 40 healthy subjects of the same age range and sex as the HAE patients who met the same exclusion criteria and were recruited at the Ophthalmology Clinic of the “Policlinico Tor Vergata”, Rome, Italy. Both eyes of each control were evaluated, and only one eye was randomly chosen for statistical analysis.

The study described has been carried out in accordance with The Code of Ethics of the World Medical Association (Declaration of Helsinki) for experiments involving humans (updated 2008). Written informed consent was obtained from patients and controls and the study was approved by the scientific ethic committee of the “Policlinico Tor Vergata”, Rome, Italy (registration number in the register of the Independent Ethics Committee of the PTV Policlinico Tor Vergata Hospital R.S.217.17).

### 2.1. Immunologic Assessment

Clinical records were registered from all HAE patients including family history, age at the onset/diagnosis, disease duration, sites of attacks, concomitant disorders and therapies, prophylactic and on-demand HAE therapies. From all the patients in the study, measurements of serum C3, C4, antigenic and functional C1INH, and C1q levels were obtained. Levels of C3 and C4 were measured using nephelometric assays (normal values 90–180 mg/dL and 10–40 mg/dL for C3 and C4, respectively), while C1q and C1INH antigen were determined with radial immunodiffusion (normal values 50–250 mg/L for C1q, 15.4–35.1 mg/dL for C1INH antigen). Functional C1INH assessment was performed from citrate plasma with chromogenic method with a normal value between 70–130% [6,30,35]. Serum levels of glucose and creatinine were added to the panel in order to confirm status of normal glycemic homeostasis and renal function: normal values were 70–99 mg/dL and 0.7–1.2 mg/dL for glucose and creatinine, respectively.

From each patient, laboratory assays were performed on the same day of the ophthalmological examination at the laboratory of the “Policlinico Tor Vergata”, Rome, Italy.

### 2.2. Ophthalmological Assessment

All subjects underwent best-corrected visual acuity (BCVA), intraocular pressure (IOP), and visual field (VF). BCVA was measured using a standard LogMAR eye chart according to the Early Treatment of Diabetic Retinopathy Study (ETDRS) protocol [36]. IOP was measured by using Goldmann applanation tonometry. Mean deviation (MD), pattern standard deviation (PSD), and visual field index (VFI) were assessed using Humphrey Field Analyzer (HFA; model 750, Zeiss Humphrey Systems, Dublin, CA, USA), using the SITA-Standard program 30–2 [30,37].

Retinal thickness analysis was obtained using the SD-OCT automated segmentation software (Spectralis; Heidelberg Engineering, Heidelberg, Germany). The posterior pole (PP) scanning protocol was performed in all patients and HC, after pupil dilation with mydriatic eye drops (phenylephrine 10% + tropicamide 0.5%, Visumidriatic Fenilefrina, Visufarma). Images were acquired using the image alignment eye-tracking software software (6.15.7.0, Heidelberg Engineering, Heidelberg, Germany) to obtain volumetric retinal scans comprised of 61 single axial scans (scanning area: 30° × 25°) centered on the fovea, with a fovea-to-disc inclination of 7 degrees. To explore a putative increase in the volume of the optic nerve head, volumetric ONH scan protocol was performed in all HAE patients and controls. The peripapillary retinal nerve fiber layer (pRNFL) thickness was also obtained with SD-OCT. An internal fixation target was used because such a method is reported to have the highest reproducibility [32,37,38].

Both eyes of each participant were examined with a 6 × 6 mm scanning protocol of the macula area using the Avanti Angiovue OCT-A (Optovue XR Avanti, Fremont, CA, USA). The vessel density (VD) was then calculated using the instrument’s built-in software. We analyzed the VD of both the superficial and deep plexus of the whole image, foveal and parafoveal zone. Poor image quality according to specific criteria including low-quality index (<7), presence of blink artifacts, motion or doubling artifacts caused by poor fixation, and media opacities obscuring the view of the vasculature were considered exclusion criteria. All examinations were performed by the same expert ophthalmologist [30,32,39,40]. All OCT-A measurements were performed at the same time of the day (specifically, 3:00–5:00 p.m.) in both patients and controls [30]. 

### 2.3. Statistical Analysis

To test the normality of data sets the D’Agostino and Pearson omnibus test was used. Mean and standard deviation (SD) express normally distributed variables. Non-normally distributed variables were analyzed using median with percentile ranges. Categorical variables were presented with absolute frequencies and percentages. Continuous variables were compared using the parametric unpaired T test or the nonparametric Mann-Whitney U test when appropriate. Categorical variables were compared using the Chi-squared test or Fisher’ exact test when appropriate. The significance of any correlation was determined by Pearson correlation test or Spearman’s rank correlation coefficient where appropriate. *p* values <0.05 were considered significant. All statistical analyses were performed using GraphPad Prism version 9 (GraphPad software).

## 3. Results

Patients (*n* = 40) affected by type I HAE (52% female) came from 22 independent families [3]. A total of 80 eyes from HAE patients were evaluated. Demographic and clinical data from the study population are reported in Table 1.

The confirmation of the inheritance of HAE was based mainly on the family history while genetic testing was conducted in 22 cases (Supplementary Table S1).

The main site of acute attacks from the HAE cohort was the abdomen (75%, 30/40) while the extremities were affected in 25% (10/40) of the patients. Patients reporting headache during the acute attacks were 15% (6/40) of the cohort and only in one patient, the headache was reported to be associated with transient visual abnormalities. Seven patients (17.5%) were on danazol long-term prophylaxis at the time of the study (dose 100 mg/day).

No correlation resulted between the levels of complement components and age, sex, age at the onset, disease duration, diagnostic delay, and the number of attacks. None of HAE patients showed abnormalities of glucose and creatinine serum levels (Table 1).

### 3.1. Ophthalmological Examinations in HAE Patients 

The BCVA values of HAE patients were within the normal range in each eye and similar to those in HC (Table 1).

HAE patients showed a median MD value (−2 dB from both right and left eyes) lower than controls (MD 0.4 dB; *p* = 0.001 for both the comparisons). The median PSD from HAE patients was significantly higher than the controls (1.7 dB) in each eye (right eye: 2.2 dB, *p* = 0.002; left eye: 2 dB, *p* = 0.03) in the presence of similar VFI between controls (range 98–100%) and HAE, in both eyes (range 94–98%) (Table 1).

**Figure 1:**
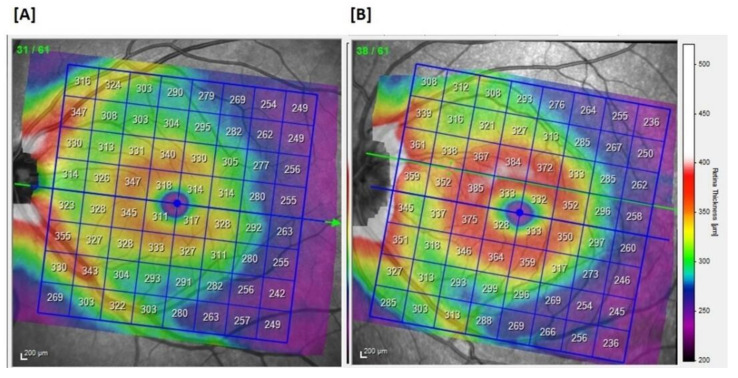
The total retinal thickness of the posterior pole. (**A**) patients. (**B**) controls.

HAE patients showed a similar mean total retinal thickness of the entire PP compared to the controls in both the eyes and both the superior and inferior hemi-fields (Figure 1, Table 2).

No correlations resulted in the HAE population between PP and the levels of complement components, sex, age at the onset, diagnostic delay, and the number of attacks. 

**Figure 2:**
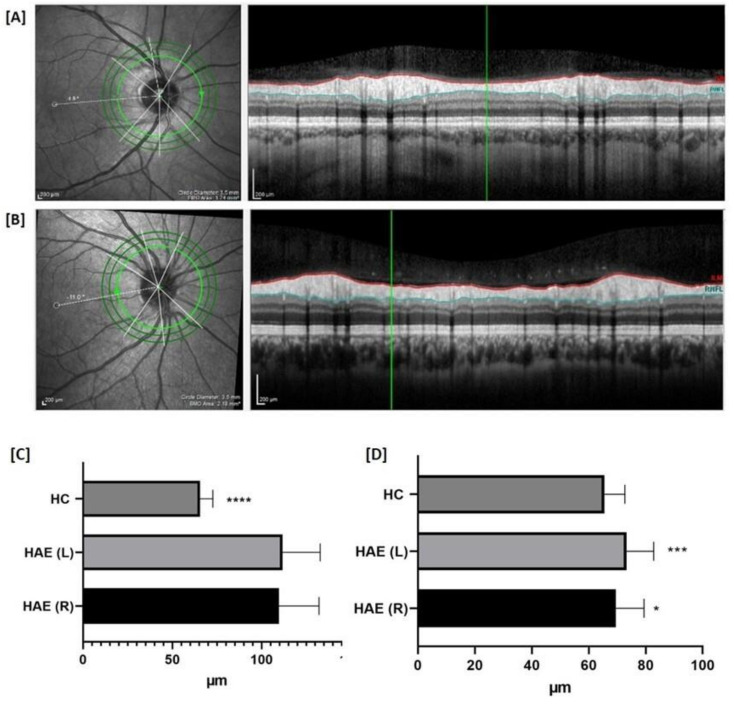
The mean thickness of peripapillary retinal nerve fiber layer (pRNFL). Representative scans of the thickness of the retinal nerve fiber layer (RNFL) from a healthy control (HC) and a patient with hereditary angioedema (HAE) are depicted in (**A**,**B**), respectively. The left image (L) shows the fundus image, which is acquired simultaneously to the optical coherence tomography (OCT) image. The right image (R) is the respective cross-sectional OCT scan of peripapillary (p)RNFL (between red and blue lines) as a grayscale image. The pRNFL thickness was higher in patients with HAE in both the right and left eyes than that in HC in nasal superior quadrants (**C**) and in temporal scans (**D**). Continuous variables were compared using the parametric unpaired T test or the nonparametric Mann-Whitney U test when appropriate. *p* < 0.05 were considered significant. * *p* < 0.05, *** *p* < 0.001, **** *p* < 0.001.

Interestingly, the pRNFL thickness was higher in patients with HAE in both the right and left eyes than that in HC in nasal superior (*p* < 0.0001 for both) and in temporal quadrants (*p* = 0.003 right, *p* = 0.0005 left) (Table 2, Figure 2). 

**Figure 3:**
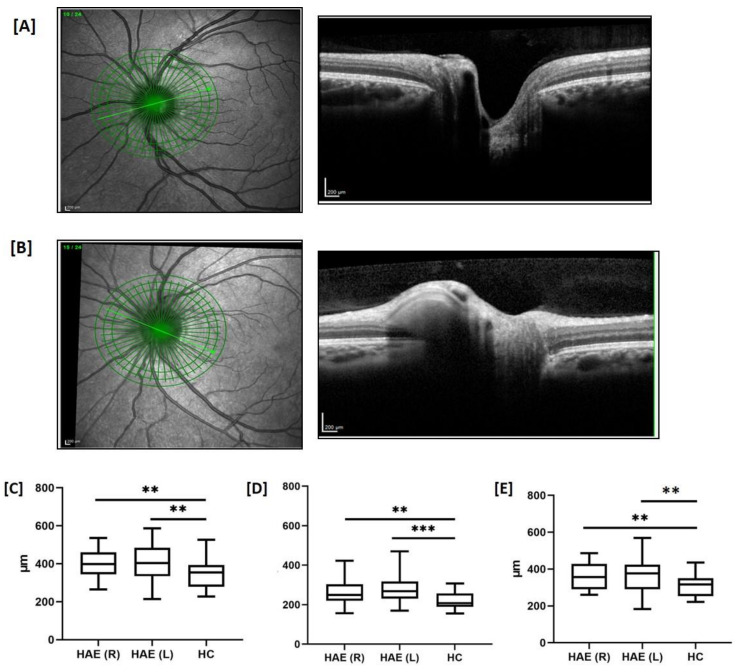
Optical coherence tomography (OCT)-derived cross-sectional images of the optic nerve head. Representative scans of the optic nerve head (ONH) from the left eye of a healthy control (HC) and a patient with hereditary angioedema (HAE) are depicted in (**A**,**B**), respectively. The left image shows the fundus image, which is acquired simultaneously to the optical coherence tomography (OCT) image. The right image is the respective cross-sectional OCT scan as a grayscale image. The green line indicates the location of the cross-section. ONH swelling occurred in the patient with HAE (**B**). ONH from patients with HAE was thicker than ONH from HC in nasal (**C**), temporal (**D**), and global (**E**) scans. R, right eyes; L, left eyes. The ONH thickness was compared between HAE and HC using the parametric unpaired T test or the nonparametric Mann-Whitney U test when appropriate. *p* < 0.05 were considered significant, ** *p* < 0.01. *** *p* < 0.001.

No correlations occurred between the pRNFL thickness and C4, C3, C1q, C1INH antigen, age, age at the onset, disease duration, diagnostic delay, and the number of attacks. In addition, no correlations resulted between pRNFL thickness and perimetric indices (MD and PSD). Representative scans of the ONH from an HC and an HAE patient are depicted in Figure 3A,B. The mean ONH thickness in HAE patients was greater in each eye than that in HC in the nasal (*p* = 0.008 left, *p* = 0.01 right), temporal (*p* = 0.0005 left, *p* = 0.003 right), temporal inferior (*p* = 0.007 left, *p* = 0.0008 right), and global (*p* = 0.005 left, *p* = 0.007 right) scans (Table 2, Figure 3C–E). 

No correlations occurred between the ONH and C4, C3, C1q, C1INH antigen and functional levels, age, age at the onset, disease duration, diagnostic delay, and the number of attacks. In addition, no correlations resulted between perimetric indices (MD and PSD) and ONH. 

**Figure 4:**
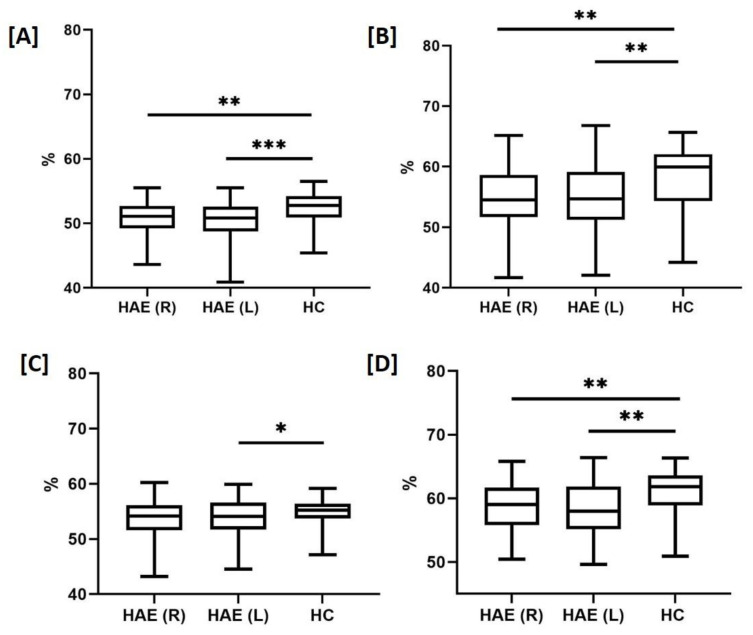
Retinal capillary plexi by optical coherence tomography angiography from the study population. Compared to healthy controls (HC), patients with hereditary angioedema (HAE) showed in both the right (R) and the left (L) eyes a lower capillary density at the both the superficial (**A**) and the deep (**B**) whole images, as well as at the superficial (**C**) and deep (**D**) parafoveal areas. The vascular density was compared between HAE and HC using the parametric unpaired T test or the nonparametric Mann-Whitney U test when appropriate. *p* < 0.05 (*) were considered significant, ** *p* < 0.01, *** *p* < 0.001.

Compared to HC, patients with HAE showed a lower capillary density in both the superficial (*p* = 0.001 left, *p* = 0.006 right) and the deep (*p* = 0.008 left, *p* = 0.004 right) whole images, as well as in the superficial (*p* = 0.03 left) and deep parafoveal (*p* = 0.007 left, *p* = 0.005 right) areas (Table 2, Figure 4A–D). Capillary density in fovea was lower in HAE than HC in all the superficial and deep scans (*p* = 0.001 for all comparisons, Table 2).

No correlations occurred between capillary density and C4, C3, C1q, C1INH antigen and functional levels, age, age at the onset, disease duration, diagnostic delay, and the number of attacks.

### 3.2. Concomitant Therapies

When stratifying patients in accordance with the danazol assumption, half of the cohort (19/40) experienced a previous (12/19) or ongoing (7/19) exposition to the long-term prophylaxis with danazol. We did not register differences between patients who have been on danazol (*n* = 19) and patients never taking danazol (*n* = 21) in all the measures (visual field, PP, ONH, and pRNFL by SD-OCT), as well as in the capillary density by OCT-A. When stratifying patients in accordance with the administration of C1INH replacement therapy for acute attacks, 20 patients were administered C1INH once in the last three months before the ophthalmological examination. We did not register differences between patients taking C1INH replacement therapy and patients who did not take it in the last three months in all the measures.

## 4. Discussion

Evidence supports that HAE may be considered as a paroxysmal permeability disorder with defective but self-limiting endothelial barrier dysfunction. Recent findings document retinal microcirculatory abnormalities in HAE patients that could precede clinical retinopathies [17,18,19,30,41,42]. Clinical evaluation of retinal and/or optic nerve head morphology and function should be performed to the early detection of potential subclinical damage. SD-OCT based methods are capable of identifying and quantifying sites of subclinical retinal and ONH alterations, and also mapping sites of changes in the retinal capillary networks [43]. No evidence to date suggests the presence of retinal or ONH damage or visual loss that potentially leads to a worse disability in HAE patients.

To our knowledge, no studies provided evidence of altered retinal or ONH thickness in HAE patients. In agreement with our results, the thickness of PP did not differ between the groups. When we analyzed the RNFL and SD-OCT findings revealed an increased RNFL thickness (nasally and temporally) from HAE patients compared to controls. Several investigations focused on the application of measurement of RNFL thickness in the early differential diagnosis among various types of optic neuropathies [44]. However, abnormalities in pRNFL can be related to changes in non-neural elements in the retina contributing to the “total” pRNFL thickness [45]. OCT-detected increments in pRNFL thickness may indicate macular edema and can be observed in primary retinal disorders causing edema, and in cases of optic nerve swelling due to a variety of pathologies, including papilledema, anterior ischemic optic neuropathy, and optic neuritis [46]. The pRNFL thickening in HAE patients may be attributable to retinal edema formation: this retinal fluid accumulation could be influenced by blood–retinal barrier permeability in addition to other mechanisms such as hydrostatic and osmotic pressure [43]. Concordantly with the pRNFL findings, ONH in HAE patients resulted thicker compared to control eyes. Such volumetric increase of the optic nerve head in HAE patients might be related to a possible temporary increase of intracranial pressure as a local manifestation of HAE. However, to the best of our knowledge, a volumetric increment of the ONH or papilledema is not present in the literature, and further investigations are needed.

According to the idea that HAE should be attributable to a self-limiting increase of endothelial permeability mediated by a variety of mediators, rather than just bradykinin [10], we previously described, evidence on retinal microvasculature changes in HAE patients without retinopathies by using OCT-A. The eyes from HAE patients had a lower mean superficial and deep whole and parafoveal density compared with healthy eyes [30,47]. In addition, it has been reported that the parafoveal area from HAE patients was thicker than in controls, suggesting a potential breakdown of the blood–retinal barrier and thus the extravascular fluid affect early the retinal thickness [30]. In turn, such an increase in retinal thickness might have reduced blood flow in the retinal plexi as we previously described.

In further investigations involving a larger HAE cohort, the analysis of the “time since last HAE episode” in each patient would probably provide useful information. Moreover, the described subclinical retinopathy in our HAE cohort may lead to chronic damage without a direct correlation with complement levels. Our data cannot definitely exclude the potential correlations between complement components and OCT/OCT-A findings because the included HAE patients were not evaluated during an acute attack (patients in remission) and also their status of complement level was influenced by the chronic both prophylactic and on-demand treatment.

About the potential impact of therapies on retinal status, we analyzed OCT and OCT-A parameters in accordance with the danazol assumption: no differences occurred between patients in all the measures [48,49]. The same resulted when HAE patients were stratified in accordance with the administration of C1INH replacement therapy for acute attacks.

## 5. Conclusions

Our data provided evidence on structural and vascular retinal changes in HAE. An increased thickness in retinal structures consistent with a fluid component within RNFL, such as the parafoveal edema, likely supports the idea of subclinical edema formation at the retina and ONH level. These early structural and microvascular changes revealed by SD-OCT and OCT-A could precede detectable damage of neuroretinopathy [50].

Future studies are needed to give complementary information and improve our findings, including the analysis of different retinal layers, as well as the evaluation of the retinal leakage with fluorescein angiography [43,44,51,52].

The knowledge of a subclinical ocular involvement in HAE will help an immunologist to reach an early diagnosis of complications and tailored managements and will alert an ophthalmologist to seek specialty consultation of an immunologist when encountered with such cases.

## Figures and Tables

**Table 1:** Demographic and clinical parameters from the study population.

	HAE (*n* = 40)	HC (*n* = 40)
Age (years)	41.4 ± 14	42 ± 10.8
Female sex (*n*/%)	21/52	17/42
Age at the onset (years)	10.4 ± 8.6	N/A
Age at the diagnosis (years)	19.7 ± 16.4	N/A
Disease duration (years)	30.7 ± 14.3	N/A
Number of attacks ^§^	18.6 ± 24.4	N/A
Attack-free period (days) ^§§^	50.1 ± 61.7	N/A
Danazol long-term prophylaxis (*n*/%)	7/17.5	N/A
C4 (mg/dL)	9.2 ± 3.5	20 ± 3
C3 (mg/dL)	118.5 ± 37.8	100 ± 42
C1q (mg/L)	144 ± 16.9	83 ± 20
C1INH (mg/dL)	6.5 ± 2.7	24 ± 4.5
C1INH (%)	31.7 ± 10.8	N/A
Creatinine (mg/dL)	0.8 ± 0.2	N/A
Glucose (mg/dL)	87.2 ± 4.2	N/A
BCVA (logMAR)	0.01 ± 0.1 (R) 0.01± 0.1 (L)	0.013 ± 0.03
IOP (mmHg)	16.5 ± 3 (R) 16.7 ± 2.9 (L)	16 ± 3
MD (median, dB)	−2 ***(R) −2 ***(L)	0.4
PSD (median, dB)	2.2 **(R) 2 *(L)	1.7
VFI (range %)	94–98 (R, L)	98–100

HAE, hereditary angioedema; HC, healthy controls; C1INH, C1 inhibitor; BCVA, best corrected visual acuity; IOP, intraocular pressure; MD mean deviation; PSD, pattern standard deviation; VFI, visual field index; R, right eyes; L, left eyes, *n*, absolute number of patients. Continuous variables were shown using mean and standard deviation (SD) while categorical variables with absolute frequencies and percentages. ^§^ in the last 12 months; ^§§^ at the time of the study. Values from patients were compared with controls using the parametric unpaired T test or the nonparametric Mann-Whitney U test when appropriate and *p* values < 0.05 were considered significant (* *p* < 0.05, ** *p* < 0.01, *** *p* < 0.001, with the respect to control eyes).

**Table 2:** SD-OCT e OCT-A findings from the study population.

		Ophthalmological Scanning	HAE		HC
			Right Eyes (*n* = 40)	Left Eyes (*n* = 40)	Control Eyes (*n* = 40)
SD-OCT	Posterior Pole (µm)	Superior Inferior Total	291.5 ± 15.1 290.4 ± 14.4 290.9 ± 14.6	288.9 ± 15.5 288.9 ± 15.2 288.8 ± 15.3	292.9 ± 13.3 292.8 ± 12.8 292.9 ± 12.9
	ONH (µm)	Nasal superior Nasal Nasal Inferior Temporal superior Temporal Temporal inferior Global	391 ± 102.7 402 ± 78.3 ** 420.2 ± 93.8 346 ± 78.6 267.2 ± 64.6 ** 391.4 ± 89.4 *** 362.5 ± 72.6 **	420 ± 118 411.2 ± 100 ** 426 ± 109 364 ± 110 * 282.7 ± 77.3 *** 381.2 ± 102.7 ** 373.6 ± 93 **	361.4 ± 96.7 344 ± 75.8 376.6 ± 73 305.8 ± 71.7 220.7 ± 43.7 318.2 ± 59.7 311.8 ± 59.5
	RNFL (µm)	Nasal superior Nasal Nasal Inferior Temporal superior Temporal Temporal inferior Global	109.7 ± 22.5 * 82.3 ± 12.5 101.3 ± 22.8 128.8 ± 17.8 73.3 ± 9.5 *** 146.7 ± 17.8 97.1 ± 8.6	111.7 ± 21.1 * 81.7 ± 14.5 101.8 ± 27.5 124.4 ± 22.4 69.5 ± 9.9 *** 138.3 ± 24.3 95.4 ± 11.8	102.7 ± 15.5 81.1 ± 10.3 113.1 ± 21.5 120.7 ± 20.4 65.5 ± 7.2 145.8 ± 16.1 94.6 ± 8.1
OCT-A	Capillary density (%)	Deep scans Fovea Parafovea Whole image Superficial scans Fovea Parafovea Whole image	41.7 ± 6.7 58.7 ± 3.6 ** 54.9 ± 4.9 ** 22.5 ± 6.1 *** 54.9 ± 3.4 50.6 ± 2.9 **	41.3 ± 6.6 58.4 ± 4.3 ** 54.9 ± 5.8 ** 22.7 ± 6.6 *** 53.1 ± 4.2 * 49.7 ± 3.4 **	41.8 ± 9.2 60.8 ± 3.4 58.1 ± 4.8 31.1 ± 8.7 55.2 ± 2.3 52.2 ± 2.6

HAE, hereditary angioedema; HC, healthy controls; ONH, optic nerve head; RNFL, retinal nerve fiber layer. Continuous variables were shown using mean and standard deviation (SD). Values from patients were compared with controls using the parametric unpaired T test or the nonparametric Mann-Whitney U test when appropriate and *p* values < 0.05 were considered significant (* *p* < 0.05, ** *p* < 0.01, *** *p* < 0.001, with the respect to control eyes).

## Supplementary Materials

The following are available online at https://www.mdpi.com/article/10.3390/jcm10225415/s1, Table S1: Mutations in C1INH gene in the study population.
