# Supplementary material for: Subclinical Signs of Retinal Involvement in Hereditary Angioedema
Source: J Clin Med. 2021 Nov 19;10(22):5415. doi: 10.3390/jcm10225415 (PMC8618365; doi:10.3390/jcm10225415)
Supplement: Supplementary file 1 [file jcm-10-05415-s001.zip › jcm-1425214-supplementary.pdf]

Table S1. Mutations in C1INH gene in the study population.

Patient [sex]	Mutations in C1INH gene
1 [F]	c.1214T>C/L405P
2 [F]	c.1214T>C/L405P
3 [M]	c.1414C>T/R472X
4 [F]	c.1414C>T/R472X
5 [F]	c.1414C>T/R472X
6 [F]	c.1414C>T/R472X
7 [M]	c.1414C>T/R472X
8 [M]	c.376G>T/D126Y
9 [M]	c.524T>A/L175H
10 [M]	c.656G>C/R219P
11 [F]	c.delG1412
12 [F]	c.delG1412
13 [M]	c365>A/s115X
14 [F]	c365>A/s115X
15 [M]	g.5528G>A/IV3-5
16 [M]	g.5528G>A/IV3-5
17 [F]	g.5528G>A/IV3-5
18 [F]	g.5528G>A/IV3-5
19 [F]	g.5528G>A/IV3-5
20 [F]	g.5528G>A/IV3-5
21 [F]	g.5528G>A/IV3-5
22 [F]	g.5528G>A/IV3-5
